# Immune checkpoint inhibitor-induced diabetes mellitus: clinical characteristics and risk factors

**DOI:** 10.3389/fimmu.2025.1499074

**Published:** 2025-01-24

**Authors:** Mei Zhan, Qinran Long, Jinhan He, Litao Huang, Bin Wu, Haixia Xu, Li Mo, Ting Xu

**Affiliations:** ^1^ Department of Pharmacy, West China Hospital, Sichuan University, Chengdu, China; ^2^ West China School of Pharmacy, Sichuan University, Chengdu, China; ^3^ Department of Pharmacy, Institute of Metabolic Diseases and Pharmacotherapy, West China Hospital, Sichuan University, Chengdu, China; ^4^ Department of Clinical Research Management, West China Hospital, Sichuan University, Chengdu, China; ^5^ Center of Gerontology and Geriatrics, West China Hospital, Sichuan University, Chengdu, China

**Keywords:** immune checkpoint inhibitors, diabetes mellitus, immune-related adverse events, cancer, immunotherapy

## Abstract

**Background:**

Emerging evidence indicates that immune checkpoint inhibitor-induced diabetes mellitus (ICI-DM) might be more common than initially reported, and more different clinical pictures associated with ICI-DM were described.

**Objective:**

The aim of our study was to identify the clinical characteristics and possible predictive factors of ICI-DM.

**Methods:**

We conducted a retrospective review of patients who received immune checkpoint inhibitors (ICI) at West China Hospital, Sichuan University until June 2023. Patients were reviewed at death or on 7 May 2024. We applied logistic regression to study the associations between clinical characteristics and ICI-DM.

**Results:**

Our study included 8,199 participants who received ICI between October 2014 and June 2023. Among them, 1,077 patients (13.14%) developed ICI-DM according to diagnostic criteria based on guidelines. By excluding patients influenced by glucocorticoids or immunosuppressants, ICI-DM was observed in 713 of 8,199 (8.70%) patients. In all patients, hypertension, hyperlipidemia, using glucocorticoids or immunosuppressants, lung cancer, and using more than one pathway of ICI were associated with a higher risk of ICI-DM. However, the risk factors for ICI-DM in patients without the influence of glucocorticoids or immunosuppressants were only hypertension, hyperlipidemia, and pancreatic lesions. In all patients and those patients without the influence of glucocorticoids and immunosuppressants, hypertension and hyperlipidemia may increase the risk for ICI-DM.

**Conclusions:**

This large, real-world cohort demonstrates that the incidence of ICI-DM may be underestimated in previous literature. Blood glucose monitoring is needed in patients receiving ICI therapy.

**Clinical trial registration:**

https://www.chictr.org.cn, identifier ChiCTR2300075974.

## 1 Introduction

Immune checkpoint inhibitors (ICI) activate anticancer immunity by blocking immune checkpoints such as programmed cell death protein 1 (PD-1), programmed cell death ligand 1 (PD-L1), and cytotoxic T-lymphocyte antigen 4 (CTLA-4). The indications for these agents continue to expand across tumor types, tremendously improving the prognosis for patients with different types of cancers. Immune-related adverse events (irAEs) are quite different from other systemic therapies such as classical chemotherapy. IrAEs may involve any organ or system, usually with the median onset within 2–16 weeks after the ICI treatment, depending on the organ system involved (1, 2). The development of irAEs varies greatly, from early occurrence within days of ICI initiation to delayed onset up to more than 1 year after the completion of ICI therapy (3, 4). Due to the growing use of ICI in oncology, clinicians will increasingly be confronted with delayed or rare irAEs. Common irAEs, including skin toxicity, thyroid disorders, colitis, and hepatitis, are well described in the literature, but rare irAEs are less defined and without evidence-based diagnostic and management strategies (5, 6).

Immune-related endocrinopathies (ir-endocrinopathies) are one of the most common irAEs and are different from other irAEs as they are usually irreversible because endocrine deficiency usually persists (7). ICI-induced diabetes mellitus (ICI-DM) is a rare but potentially life-threatening adverse event. The incidence rate of ICI-DM reported in the literature varies from 0.2% to 1.9% (8–10). The diagnosis of ICI-DM is inconsistent according to literature reports. Research studies from a database of adverse event reports used the terms diabetic ketoacidosis (DKA) and type 1 diabetes mellitus (T1DM) to identify ICI-DM, and the plasma glucose level for diagnosing ICI-DM in retrospective studies ranged from 11.1 mmol/L to 16.0 mmol/L (8, 9, 11). ICI-DM has similarities to type 1 diabetes, but it represents a new clinical entity (12, 13). Therefore, using the diagnostic criteria of type 1 diabetes as the diagnostic criteria of ICI-DM would lead to missed diagnosis. With the increasing use of ICI in clinical practice, the incidence of ICI-DM is also growing (14). As ICI-DM is still largely unknown, we conducted a retrospective study to evaluate the clinical characteristics and risk factors of ICI-DM after ICI therapy.

## 2 Methods

### 2.1 Study design and patient selection

This retrospective cohort study of patients who received at least one dose of ICI was performed at West China Hospital, Sichuan University until June 2023. ICIs included PD-1 inhibitors (pembrolizumab, nivolumab, tislelizumab, penpulimab, camrelizumab, sintilimab, toripalimab, serplulimab, pucotenlimab, LZM009, iparomlimab), PD-L1 inhibitors (atezolizumab, durvalumab, benmelstobart, adebrelimab, envafolimab, tagitanlimab), CTLA-4 inhibitors (ipilimumab, SHR8068, IBI310, quavonlimab, porustobart), TIGIT inhibitors (ociperlimab, vibostolimab, IBI939), lymphocyte activation gene 3 (LAG-3) inhibitor (LBL-007), B and T lymphocyte attenuator (BTLA) inhibitor (tifcemalimab), dual blockade of PD-1 and LAG-3 (MK4208A), dual blockade of PD-L1/TGF-β (SHR1701), dual blockade of PD-1/CTLA-4 (cadonilimab), dual blockade of CLDN18.2/CD3 (IBI389), and dual blockade of CD47/PD-L1 (IBI322). Patients were assessed at death or on 7 May 2024, whichever occurred first. The diagnostic criteria for ICI-DM were based on the diabetes diagnostic criteria issued by the American Diabetes Association (ADA) and the management guidelines for immunotherapy-related hyperglycemia issued by the National Comprehensive Cancer Network (NCCN) (15, 16). The criteria for the diagnosis of ICI-DM were as follows: 1) fasting plasma glucose (FPG) ≥126 mg/dL (7.0 mmol/L), 2h-PG ≥200 mg/dL (11.1 mmol/L) during the oral glucose tolerance test (OGTT), random plasma glucose ≥200 mg/dL (11.1 mmol/L), or glycated hemoglobin A1c (HbA1c) ≥6.5% (48 mmol/mol) in a patient without diabetes before receiving ICI and 2) FPG or a random plasma glucose >250 mg/dL (13.9 mmol/L) in a patient with a history of type 2 diabetes. Diagnosis requires at least two abnormal test results. This study was approved by the Biomedical Ethics Review Committee of West China Hospital, Sichuan University (2023 Review [No. 1064]).

### 2.2 Data collection

Basic information such as age, sex, and findings of available laboratory tests through a big data platform was extracted at West China Hospital of Sichuan University. Details of ICI therapy and the combination of medications were collected from electronic medical records. Because elevated plasma glucose is not a common adverse reaction of inhaled and topical glucocorticoids, only oral or intravenous preparations such as dexamethasone, methylprednisolone, prednisone, and hydrocortisone, but not external or inhaled preparations, were included. Immunosuppressants included oral preparations such as mycophenolate, tacrolimus, and sirolimus. Plasma glucose and C-peptide levels were measured in the clinical laboratories at West China Hospital, Sichuan University. The following data were captured at baseline: age, sex, primary tumor site, type of ICI, treatment start date, history of diabetes or abnormal glucose tolerance, plasma glucose level before receiving ICI, and smoking or drinking status. Pancreatic lesions included pancreatic cancer, pancreatitis, pancreatic metastasis, and imaging abnormalities of the pancreas.

### 2.3 Statistical analysis

Patient characteristics were reported as medians and interquartile ranges (IQRs) for continuous variables that were not normally distributed, as means with standard deviations for normally distributed data, and as frequencies and percentages for categorical variables. In the regression analysis, we converted age into a categorical variable: under 18 years, 18–60 years, 60.1–80 years, and above 80 years (17). Univariate and multivariable logistic regression analyses were performed to determine those variables significantly contributing to ICI-DM using SPSS version 29.0 software. Predictor variables for multivariable regression were prespecified based on clinical experience and significance in univariable regression. Variance inflation factors (VIFs) were computed to assess for multicollinearity by collinear diagnosis. Indicators as independent variables with *p <*0.20 were used in the univariate logistic regression analysis, and ICI-DM status as the dependent variable (assigned value: occurrence = 1, non-occurrence = 0) was used to perform multivariable logistic regression analysis. The results of the multivariate logistic analysis were expressed as adjusted odds ratios (ORs) with their 95% confidence intervals (95% CIs), and *p <*0.05 was considered to indicate statistical significance.

Regarding the handling of missing data, we first attempted to complete the data where possible. If the missing rate was less than 20%, considering the potential bias introduced by data imputation, we opted to directly exclude samples with missing data from the analysis.

## 3 Results

### 3.1 Clinical characteristics of ICI-DM

**Figure 1:**
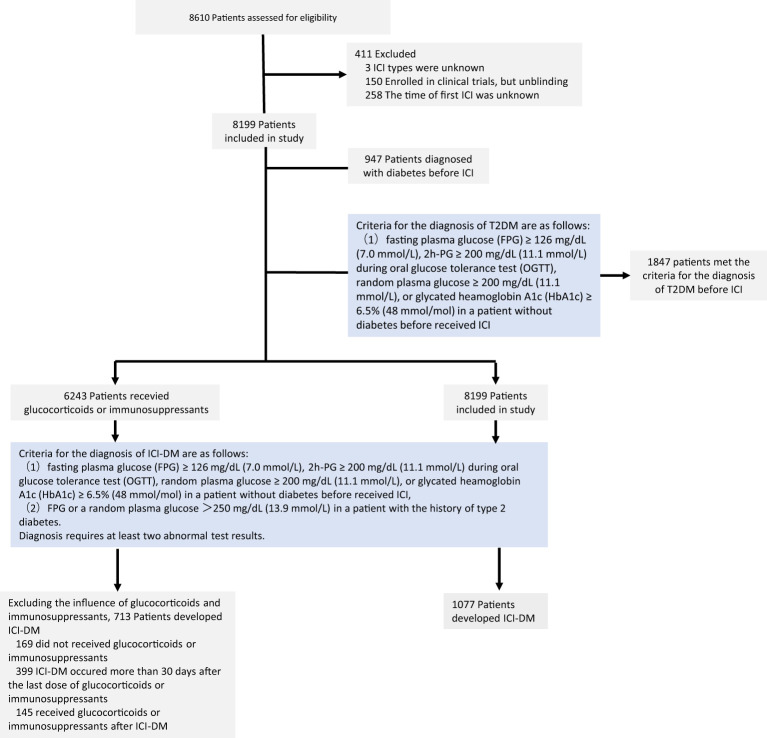
Flowchart of patients receiving immune checkpoint inhibitors: screening, enrollment, and results. ICI, immune checkpoint inhibitor; T2DM, type 2 diabetes mellitus; ICI-DM, immune checkpoint inhibitor-induced diabetes mellitus.

Initially, a total of 8,610 patients received ICI therapy between October 2014 and June 2023. In total, 8,199 patients remained in the study cohort after excluding 411 patients who lacked key information such as the time of first ICI use (**Figure 1**). These 8,199 patients had a median age of 58.80 years, and 25.92% were women. Before receiving ICI treatment, the plasma glucose of 1,847 patients met the criteria for the diagnosis of type 2 diabetes, but only 947 patients were diagnosed with diabetes. Three patients were diagnosed with abnormal glucose tolerance before ICI therapy, and two of these patients met the criteria for the diagnosis of diabetes. When their FPG or random plasma glucose increased to >250 mg/dL (13.9 mmol/L), it was defined as ICI-DM. ICI-DM was observed in 141 of 1,847 (7.63%) patients. The median time from ICI initiation to ICI-DM was 177 (IQR 57.50–383.50) days. There were 6,351 patients who had no prior diagnosis of diabetes or abnormal glucose tolerance before treatment, and their plasma glucose did not meet the diagnostic criteria for diabetes before ICI treatment. There were 936 patients (14.74%) who developed ICI-DM during the ICI treatment. The median time of newly diagnosed ICI-DM was 124 (IQR 48.50–325.00) days after the first treatment cycle. The baseline characteristics are shown in **Table 1**.

**Table 1:** Baseline patient demographics and clinical characteristics.

	All *n*	All patients			Patients without the influence of glucocorticoids and immunosuppressants		
		ICI-DM, *n* (%)	Non-ICI-DM, *n* (%)	*p*-value	ICI-DM, *n* (%)	Non-ICI-DM, *n* (%)	*p*-value
Total	8,199	1,077 (13.14)	7,122 (86.86)		713 (8.70)	7,486 (91.30)	
Age (years), median [IQR]	58.80 [52.00-67.10]	59.90[54.10-67.60]	58.50 [50.80–67.00]	0.234	60.95 [54.75–68.10]	58.00 [50.80–66.55]	0.034
Sex				0.025			0.334
Female	2,125	249 (23.12)	1,876 (26.34)		174 (24.40)	1,951 (26.06)	
Male	6,074	828 (76.88)	5,246 (73.66)		539 (75.60)	5,535 (73.94)	
Comorbidities							
Tobacco use	3,296	464 (43.08)	2,832 (39.76)	0.096	301 (42.22)	2,995 (40.01)	0.366
Alcohol use	2,399	327 (30.36)	2,072 (29.09)	0.6	214 (30.01)	2,185 (29.19)	0.791
Diabetes or abnormal glucose tolerance	1,848	141 (13.09)	1,707 (23.97)	<0.001	111 (15.57)	1,737 (23.20)	<0.001
Hypertension	1,696	271 (25.16)	1,425 (20.01)	<0.001	178 (24.96)	1,518 (20.28)	0.003
Hyperlipidemia	343	72 (6.69)	271 (3.81)	<0.001	47 (6.59)	296 (3.95)	<0.001
Pancreatic lesions	595	80 (7.43)	515 (7.23)	0.816	62 (8.70)	533 (7.12)	0.122
Using glucocorticoids or immunosuppressants	6,243	908 (84.31)	5,335 (74.91)	<0.001	544 (76.30)	5,699 (76.13)	0.92
Primary cancer				<0.001			0.05
Lung	3,112	504 (46.80)	2,608 (36.62)		302 (42.36)	2,810 (37.54)	
Liver	801	77 (7.15)	724 (10.17)		57 (7.99)	744 (9.94)	
Gastric	700	74 (6.87)	626 (8.79)		51 (7.15)	649 (8.67)	
Esophageal	694	91 (8.45)	603 (8.47)		61 (8.56)	633 (8.46)	
Multiple primary tumors	133	9 (0.84)	125 (1.76)	0.032	6 (0.84)	127 (1.70)	0.091
Other	2,759	322 (29.90)	2,437 (34.22)		236 (33.10)	2,523 (33.70)	
Immune checkpoint targets				0.07			0.111
PD-1	7,264	927 (86.07)	6,337 (88.98)		615 (86.26)	6,649 (88.82)	
PD-L1	642	93 (8.64)	549 (7.71)		62 (8.70)	580 (7.75)	
CTLA-4	1	0 (0.00)	1 (0.01)		0 (0.00)	1 (0.01)	
More than one target	292	57 (5.29)	235 (3.30)		36 (5.05)	256 (3.42)	

IQR, interquartile ranges; ICI-DM, immune checkpoint inhibitor-induced diabetes mellitus; PD-1, programmed cell death protein 1; PD-L1, programmed cell death ligand 1; CTLA-4, cytotoxic T-lymphocyte antigen 4.

In the study cohort, 6,243 patients (76.14%) received glucocorticoids or immunosuppressants such as mycophenolate mofetil and tacrolimus during the follow-up period. Among them, 4,441 patients had a cumulative glucocorticoid or immunosuppressant duration in the hospital exceeding 10 days. The reasons for using glucocorticoids included pretreatment with antitumor drugs, radiation pneumonitis, treatment for irAEs, and so on. Patients used immunosuppressants after organ transplantation surgery and if they had severe irAEs.

Glucocorticoids and immunosuppressants could be associated with hyperglycemia, and there was a lack of indicators to distinguish between ICI and other drugs that induced hyperglycemia. We extracted patient medication information to determine the cause of new-onset diabetes or worsening of preexisting diabetes. Among the 1,077 patients with ICI-DM, 169 patients did not use glucocorticoids and immunosuppressants, 145 patients received glucocorticoids or immunosuppressants after the time of new-onset diabetes, and 399 patients received glucocorticoids or immunosuppressants more than 30 days before the onset of new diabetes. We considered newly diagnosed diabetes and the worsening of preexisting diabetes of these patients as not related to glucocorticoids or immunosuppressants. Excluding patients influenced by glucocorticoids or immunosuppressants, ICI-DM was observed in 713 of 8,199 (8.70%) patients.

According to the NCCN guidelines, C-peptide should be measured to distinguish insulin resistance (T2DM) or steroid-related hyperglycemia from immune checkpoint inhibitor-induced type 1 diabetes mellitus (ICI-T1DM). C-peptide testing included fasting serum C-peptide, 1-h serum C-peptide, 2-h serum C-peptide, and 3-h serum C-peptide. Only 43 patients had a record of C-peptide testing after ICI therapy, of which 37 patients had abnormal C-peptide levels. Among them, five patients had a low level of C-peptide after ICI treatment, but their plasma glucose did not meet the criteria for ICI-DM diagnosis. There were 7,358 patients (89.7%) who had testing results of FPG after ICI treatment, but only 852 patients (10.4%) had testing results of HbA1c after ICI treatment. Only one patient tested for insulin autoantibodies (IAAs) and antiglutamic acid decarboxylase antibody (GADA) after ICI treatment, who was defined as ICI-DM. The level of GADA was 0.47 U/mL and the IAAs were positive. For more details, see **Table 2**. In our study, there were only five patients (0.06%) who were newly diagnosed with T1DM and six patients (0.07%) who were newly diagnosed with DKA after ICI treatment. Among these ICI-DM patients, 165 patients (15.32%, 165/1,077) had records of prescribed insulin after ICI treatment and 33 patients (3.06%, 33/1,077) received insulin before ICI treatment in the HIS system. The data of the patients who used insulin after ICI are summarized in **Appendix 1** in **Supplementary Material**.

**Table 2:** Laboratory data before and after ICI treatment.

	Before ICI treatment						After ICI treatment					
	All patients (*n* = 8,199)		ICI-DM (*n* = 1077)		ICI-DM^a^ (*n* = 713)		All patients (*n* = 8199)		ICI-DM (*n* = 1,077)		ICI-DM^a^ (*n* = 713)	
	*N*	Value	*N*	Value	*N*	Value	*N*	Value	*N*	Value	*N*	Value
Fasting C-peptide median [IQR], nmol/L	94	0.690 [0.547–0.916]	10	0.659 [0.381–1.030]	8	0.637 [0.381–1.480]	43	0.315 [0.008–0.700]	32	0.268 [0.007–0.634]	22	0.260 [0.007–0.692]
C-peptide 1 h postprandial, median [IQR], nmol/L	19	1.720 [1.185–2.495]	0	/	0	/	4	1.290 [0.007–1.590]	4	1.290 [0.007–1.590]	1	1.59
C-peptide 2 h postprandial median [IQR], nmol/L	56	2.265 [1.433–3.035]	3	0.894 [0.859–1.712]	3	0.894 [0.859–1.712]	15	1.170 [0.493–2.070]	12	1.470 [0.129–2.070]	8	1.470 [0.220–2.050]
C-peptide 3 h postprandial median [IQR], nmol/L	13	1.650 [1.050–2.380]	0	/	0	/	1	0.048	1	0.048	1	0.048
Fasting plasma glucose median [IQR], mmol/L	7,433	5.410 [4.940–6.083]	938	5.370 [4.870–6.140]	629	5.390 [4.870–6.180]	7,358	5.280 [4.780–6.110]	1,076	5.810 [5.050–7.240]	711	5.900 [5.130–7.490]
HbA1c median [IQR], %	992	6.30 [5.75–7.45]	119	6.70 [6.00–7.50]	85	6.60 [5.95–7.50]	852	6.30 [5.80–7.60]	267	6.80 [6.10–8.30]	194	7.00 [6.20–8.35]

IQR, interquartile range; ICI-DM, immune checkpoint inhibitor-induced diabetes mellitus; ICI, immune checkpoint inhibitor; HbA1c, glycosylated hemoglobin, type A1C.

a Patients without the influence of glucocorticoids and immunosuppressants.

### 3.2 Factors associated with ICI-DM

We conducted a logistic regression analysis by including both patients without the influence of glucocorticoids or immunosuppressants and all patients. Based on the results of univariate logistic regression, sex, tobacco use, diabetes or abnormal glucose tolerance, hypertension, hyperlipidemia, using glucocorticoids or immunosuppressants, primary cancer, multiple primary tumors, and immune checkpoint targets should be included in multivariate logistic regression for all patients. Meanwhile, age, diabetes or abnormal glucose tolerance, hypertension, hyperlipidemia, primary cancer, pancreatic lesions, primary cancer, multiple primary tumors, and immune checkpoint targets should be included in multivariate logistic regression for patients without the influence of glucocorticoids or immunosuppressants. The results of univariate logistic regression are shown in **Table 1**.

The collinearity diagnosis showed that there was no serious multicollinearity requiring correction in our model. In the multivariate analysis, patients with ICI-DM were significantly more likely to be treated with glucocorticoids or immunosuppressants (OR 1.974 [95% CI 1.616 to 2.409], *p <* 0.001) among all patients. The combination with hypertension or hyperlipidemia may increase the risk of ICI-DM among all patients and patients without the influence of glucocorticoids or immunosuppressants. In the two groups of patients, those with a history of diabetes or abnormal glucose tolerance seemed to have a lower risk of ICI-DM. Each of the following was independently associated with the risk of ICI-DM only among all patients: lung cancer (OR = 1.273 [95% CI 1.070 to 1.514], *p* = 0.007), blockade of two or more immune checkpoints (OR = 1.522 [95% CI 1.096 to 2.113], *p* = 0.012), and male sex (OR = 1.262 [95% CI 1.042 to 1.528], *p* = 0.017). For patients without the influence of glucocorticoids, pancreatic lesions may increase the risk of ICI-DM (OR = 1.361 [95% CI 1.013 to 1.829], *p* = 0.041). The results of multivariate regression are shown in **Table 3**.

**Table 3:** Logistic multivariate regression analysis of risk factors for ICI-DM.

	All patients				Patients without the influence of glucocorticoids and immunosuppressants			
	*p*-value	OR	95% CI		*p*-value	OR	95% CI	
			Lower limit	Upper limit			Lower limit	Upper limit
Sex	0.017	1.262	1.042	1.528				
Age								
<18					0.200			
18–60					0.593	1.732	0.232	12.958
60.1–80					0.485	2.051	0.273	15.382
>80					0.726	1.457	0.178	11.947
Tobacco use	0.988	1.001	0.850	1.179				
Diabetes or abnormal glucose tolerance	<0.001	0.246	0.194	0.313	<0.001	0.376	0.291	0.487
Hypertension	<0.001	1.396	1.183	1.647	0.013	1.284	1.054	1.565
Hyperlipidemia	<0.001	1.664	1.241	2.232	<0.001	1.672	1.192	2.345
Pancreatic lesions					0.041	1.361	1.013	1.829
Using glucocorticoids or immunosuppressants	<0.001	1.974	1.616	2.409				
Primary cancer								
Other	0.004				0.458			
Lung	0.007	1.273	1.070	1.514	0.673	1.044	0.854	1.277
Liver	0.315	0.859	0.638	1.156	0.420	0.875	0.631	1.212
Gastric	0.449	0.897	0.676	1.189	0.271	0.830	0.595	1.157
Esophageal	0.942	1.010	0.765	1.335	0.649	0.927	0.668	1.285
Multiple primary tumors	0.171	0.614	0.306	1.234	0.169	0.555	0.240	1.284
Immune checkpoint targets								
PD-1	0.076				0.483			
PD-L1	0.335	1.132	0.880	1.456	0.475	1.114	0.829	1.495
CTLA-4	1.000	0.000	0.000		1.000	0.000	0.000	
More than one target	0.012	1.522	1.096	2.113	0.147	1.342	0.902	1.996

ICI-DM, immune checkpoint inhibitor-induced diabetes mellitus; OR, odds ratio; CI, confidence interval; PD-1, programmed cell death protein 1; PD-L1, programmed cell death ligand 1; CTLA-4, cytotoxic T-lymphocyte antigen 4.

## 4 Discussion

This review represents the largest number of confirmed ICI-DM cases in the real world, with the inclusion of the diagnostic criteria from the ADA and NCCN. A key finding of our work is that the incidence of ICI was significantly higher than that reported in the literature. This difference may be due to the following reasons: firstly, previous studies focused mainly on ICI-induced T1DM, and diabetic ketoacidosis (DKA) was often the first manifestation in case reports. Patients who were hyperglycemic but without DKA might lead to missed diagnosis (14). Most cases of ICI-DM reported were insulin-dependent diabetes, which was similar to T1DM. However, other cases had a phenotype close to type 2 diabetes or did not require lifelong insulin therapy (18–20). More milder cases have recently been reported, suggesting that ICI-DM should be considered as a new entity of DM (13, 18). Limited data are available regarding ICI-DM, and heterogeneous diagnostic criteria have been applied (21, 22). Due to the wide variety of ICI-DM and as no clear diagnostic criteria existed, relatively mild forms of ICI-DM were unable to be identified (23, 24). Secondly, patients remain at risk for ICI-DM after discontinuation of ICI, and the mean time of onset of ICI-DM ranged from 7 to 25 weeks after the initiation of ICI treatment (25). Even if hyperglycemia does not occur during the ICI treatment, ICI-DM may still occur after discontinuation of ICI treatment. Because ICI-DM is a rare, late-onset irAE, its identification in clinical trials is difficult. The reported incidence rate of ICI-DM in clinical trials is significantly lower than in clinical practice (26). Finally, there is an underuse of guideline-recommended care in patients receiving ICI treatment. The reported proportion of patients with their blood glucose monitored in every treatment cycle was only 34.8% in China (27). In addition, the early symptoms of diabetes might be ignored, leading to an underestimation of the incidence rate of ICI-DM.

Another finding in our study is that we compared the risk factors for ICI-DM among different populations. Patients with cancer often receive glucocorticoids and immunosuppressants during treatment. Regarding the wide variety of ICI-DM, if taking glucocorticoids and immunosuppressants at the time of the onset of hyperglycemia were excluded, the incidence of ICI will be underestimated. Because our sample size is large enough, we can analyze multiple risk factors in both all patients and patients without the influence of glucocorticoids or immunosuppressants. The results from previous studies investigating the association between risk factors and ICI-DM were conflicting. Chen’s study demonstrated that younger age and preexisting non-T1DM diabetes increased the incidence of ICI-T1DM, while prior use of immunosuppressive medications was associated with a lower incidence of ICI-T1DM (8). On the other hand, Takada’s study demonstrated that the incidence of ICI-T1DM was significantly higher in women with melanoma (28). Chan’s study even demonstrated that men had a significantly higher risk of ICI-DM (29). Our study showed no significant difference in overall ICI-DM between women and men in the patient group without the influence of glucocorticoids and immunosuppressants, but men had an increased risk of developing ICI-DM in all patients. In both all patients and patients without the influence of glucocorticoids and immunosuppressants, hypertension and hyperlipidemia might increase the incidence of ICI-DM, while the incidence of ICI-DM was significantly lower in patients with diabetes or abnormal glucose tolerance. It can be partially explained by the following fact: prediabetes is always associated with dyslipidemia and hypertension (30). It is difficult to distinguish diabetes from ICI-DM, so a patient with hypertension or hyperlipidemia has a higher incidence of ICI-DM.

There are several limitations to our study that warrant consideration. Firstly, this is a retrospective, single-center study and, therefore, may not be generalizable. Retrospective studies rely on existing clinical databases, but these databases are not specifically designed for clinical research, so in most cases, some data may inevitably be missing. In our study, it is also essential to measure C-peptide levels to differentiate between insulin resistance (such as T2DM or steroid-related hyperglycemia) and immune checkpoint inhibitor-induced type 1 diabetes mellitus (ICI-T1DM). However, only 149 patients (1.81%) had a C-peptide test record following ICI therapy, of which 69 patients had abnormal C-peptide levels. This low testing rate weakens the study’s ability to make definitive conclusions about the mechanisms underlying hyperglycemia in this patient population. ICI-DM is a rare irAE, and the available data are mostly limited to case reports and small series. Our study included nearly 10 years of data, and a large sample size of patients received ICI therapy at a tertiary hospital. Therefore, it still can provide important information on the epidemiology and possible predictive factors of ICI-DM. Secondly, new-onset hyperglycemia or the worsening of preexisting diabetes during ICI therapy can be attributed to many factors, including glucocorticoids or other drugs that may cause hyperglycemia, stress-induced hyperglycemia, and pancreatogenic diabetes. We only extracted medication information from hospital medical records, and drugs used outside the hospital that may increase plasma glucose were overlooked. We did not calculate the dosage when analyzing the effects of glucocorticoids or immunosuppressants on blood glucose. With the emergence of the multifaceted nature of ICI-DM, it has become increasingly difficult to distinguish ICI-DM from other types of diabetes. In addition, the ADA diagnostic criteria for diabetes mellitus do not capture the clinical heterogeneity of ICI-DM. Considering the high rate of missed diagnosis of diabetes in China (31, 32), patients who meet the diagnostic criteria for diabetes but without diabetes diagnosis are treated as diabetes patients in our study. Tumors, stress state, antitumor treatments, and other medications such as steroids may cause hyperglycemia. Therefore, hyperglycemia and diabetes may occur during the treatment of cancer. It led to the misdiagnosis of diabetes before and after ICI in our study. Therefore, ICI-DM in our study may include other types of diabetes, and the ICI-DM incidence might be overestimated. Finally, the analysis of the influence of diabetes on ICI-DM was affected by many factors. Due to the lack of accepted diagnostic criteria, various studies used different definitions for ICI-DM, especially for patients with diabetes before ICI treatment. In our study, patients with diabetes had more stringent criteria for the diagnosis of ICI-DM, which may be one of the reasons that patients with diabetes may have a reduced risk of ICI-DM. This may also be attributed to the overdiagnosis of diabetes before ICI treatment. We diagnosed ICI-DM after ICI treatment based on whether blood glucose meets the diagnostic criteria for diabetes, rather than the diagnosis in the medical record. To ensure consistency in diagnostic criteria before and after ICI treatment and considering that research shows that the missed diagnosis rate of diabetes is very high, we treat those who meet the diagnostic criteria for diabetes but have not been diagnosed with diabetes as diabetes before ICI. However, this might indeed mistake non-diabetics as diabetic, so we conducted a supplementary analysis. If only patients diagnosed with diabetes were considered to have diabetes before ICI treatment, the incidence of ICI-DM was 17.25% (1,414/8,199), and univariate logistic regression showed that diabetes might increase the risk of ICI-DM (OR = 1.381 [95% CI 1.169 to 1.631], *p* < 0.001) in all patients. The results of the logistic regression analysis are shown in **Appendix 2** in **Supplementary Material**. Therefore, it cannot be concluded that patients with diabetes may have a reduced risk of ICI-DM.

## 5 Conclusion

In conclusion, we reported the incidence of ICI-DM among those receiving ICI, which considerably exceeds the rates reported in previous literature. Patients receiving ICI therapy need blood glucose monitoring, especially those with hyperlipidemia or hypertension.

## Data Availability

The raw data supporting the conclusions of this article will be made available by the authors, without undue reservation.
