# Supplementary material for: Immune checkpoint inhibitor-induced diabetes mellitus: clinical characteristics and risk factors
Source: Front Immunol. 2025 Jan 24;16:1499074. doi: 10.3389/fimmu.2025.1499074 (PMC11802519; doi:10.3389/fimmu.2025.1499074)
Supplement: Supplementary file 2 [file Table2.docx]

| Appendix 2 Logistic Regression Analysis of Risk Factors for ICI-DM | | | | | | | | |
| --- | --- | --- | --- | --- | --- | --- | --- | --- |
| Characteristic | Univariable Logistic Regression | | | | Multivariable Logistic Regression | | | |
|  | P | OR | 95%CI | | P | OR | 95%CI | |
|  |  |  | lower limit | upper limit |  |  | lower limit | upper limit |
| Sex | ＜0.001 | 1.257 | 1.097 | 1.440 |  |  |  |  |
| age |  |  |  |  |  |  |  |  |
| ＜18 | 0.056 |  |  |  | 0.670 |  |  |  |
| 18-60 | 0.184 | 2.653 | 0.629 | 11.181 | 0.335 | 2.042 | 0.478 | 8.720 |
| 60.1-80 | 0.130 | 3.039 | 0.721 | 12.809 | 0.304 | 2.145 | 0.501 | 9.180 |
| ＞80 | 0.223 | 2.526 | 0.569 | 11.208 | 0.330 | 2.115 | 0.468 | 9.555 |
| Tobacco use | 0.074 | 1.112 | 0.990 | 1.249 |  |  |  |  |
| Diabetes* | ＜0.001 | 1.381 | 1.169 | 1.631 | 0.049 | 1.193 | 1.001 | 1.421 |
| Hypertension | ＜0.001 | 1.459 | 1.277 | 1.666 | ＜0.001 | 1.307 | 1.134 | 1.506 |
| Hyperlipidemia | ＜0.001 | 2.049 | 1.611 | 2.605 | ＜0.001 | 1.713 | 1.337 | 2.195 |
| Pancreatic Lesions | 0.004 | 1.347 | 1.098 | 1.652 | 0.003 | 1.379 | 1.115 | 1.704 |
| Using glucocorticoids or immunosuppressants | ＜0.001 | 2.038 | 1.741 | 2.386 | ＜0.001 | 1.983 | 1.686 | 2.332 |
| Primary cancer |  |  |  |  |  |  |  |  |
| Other | ＜0.001 |  |  |  | ＜0.001 |  |  |  |
| Lung | ＜0.001 | 1.411 | 1.234 | 1.614 | ＜0.001 | 1.343 | 1.159 | 1.558 |
| Liver | 0.903 | 0.987 | 0.794 | 1.226 | 0.149 | 1.181 | 0.942 | 1.481 |
| Gastric | 0.143 | 0.836 | 0.657 | 1.063 | 0.130 | 0.829 | 0.649 | 1.057 |
| Esophageal | 0.595 | 0.939 | 0.744 | 1.185 | 0.730 | 0.958 | 0.751 | 1.223 |
| Multiple primary tumors | 0.175 | 0.685 | 0.396 | 1.183 | 0.119 | 0.644 | 0.371 | 1.119 |
| Immune checkpoint targets |  |  |  |  |  |  |  |  |
| PD-1 | 0.007 |  |  |  | 0.042 |  |  |  |
| PD-L1 | 0.441 | 1.086 | 0.880 | 1.340 | 0.505 | 0.928 | 0.745 | 1.156 |
| CTLA-4 | 1.000 | 0.000 | 0.000 |  | 1.000 | 0.000 | 0.000 |  |
| More than one targets | 0.001 | 1.612 | 1.227 | 2.118 | 0.006 | 1.474 | 1.115 | 1.948 |

* Diabetes: Diagnosis of diabetes before ICI treatment

Abbreviations: IQR: interquartile ranges, ICI-DM: immune checkpoint inhibitors induced diabetes mellitus; PD-1: programmed cell death protein 1; PD-L1: programmed cell death ligand 1; CTLA-4 cytotoxic T-lymphocyte antigen 4.
